# Author Correction: A glomerulus-on-a-chip to recapitulate the human glomerular filtration barrier

**DOI:** 10.1038/s41467-019-12177-7

**Published:** 2019-10-21

**Authors:** Astgik Petrosyan, Paolo Cravedi, Valentina Villani, Andrea Angeletti, Joaquin Manrique, Alessandra Renieri, Roger E. De Filippo, Laura Perin, Stefano Da Sacco

**Affiliations:** 1 GOFARR Laboratory for Organ Regenerative Research and Cell Therapeutics in Urology, Saban Research Institute, Division of Urology, Children’s Hospital Los Angeles, Los Angeles, CA USA; 2 Division of Nephrology, Icahn School of Medicine at Mount Sinai, New York, NY USA; 3 Department of Experimental, Diagnostic and Specialty Medicine, Nephrology, Dialysis and Renal Transplant Unit, S. Orsola-Malpighi Hospital, University of Bologna, Bologna, Italy; 4 Nephrology Service, Complejo Hospitalario de Navarra, Pamplona, Spain; 5 Genetica Medica, Azienda Ospedaliera Universitaria Senese, Siena, Italy; 6 Department of Urology, Keck School of Medicine, University of Southern California, Los Angeles, CA USA

**Keywords:** Lab-on-a-chip, Glomerulus

Correction to: *Nature Communications* 10.1038/s41467-019-11577-z, published online 13 August 2019.

This Article omits a declaration from the Competing Interests statement, which should have included the following: ‘R.D.F., L.P. and S.D.S. are inventors on a U.S. provisional patent relating to Glomerulus-on-a-Chip, submitted by Children’s Hospital Los Angeles. The other authors declare no competing interests.’

These errors have been corrected in the PDF and HTML versions of the article.

