# Supplementary material for: A glomerulus-on-a-chip to recapitulate the human glomerular filtration barrier
Source: Nat Commun. 2019 Aug 13;10:3656. doi: 10.1038/s41467-019-11577-z (PMC6692336; doi:10.1038/s41467-019-11577-z)
Supplement: Supplementary file 1 — Supplementary Information [file 41467_2019_11577_MOESM1_ESM.pdf]

Supplementary Information

A glomerulus-on-a-chip to recapitulate the human glomerular filtration barrier

Petrosyan et al.

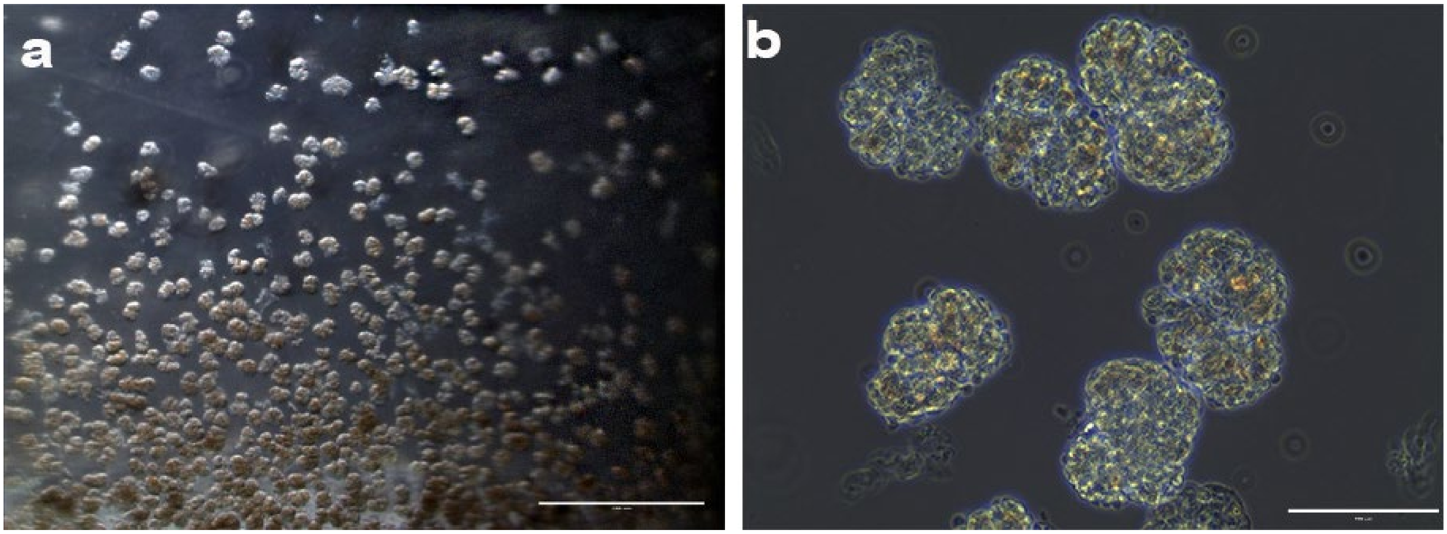

Supplementary Figure 1: Human glomeruli

a-b. Representative bright field imaging of human glomeruli immediately upon isolation confirming yield and purity of the isolation process (as described in the material and methods). Scale bar= 1000 μm (**a**), scale bar= 100 μm (**b**).

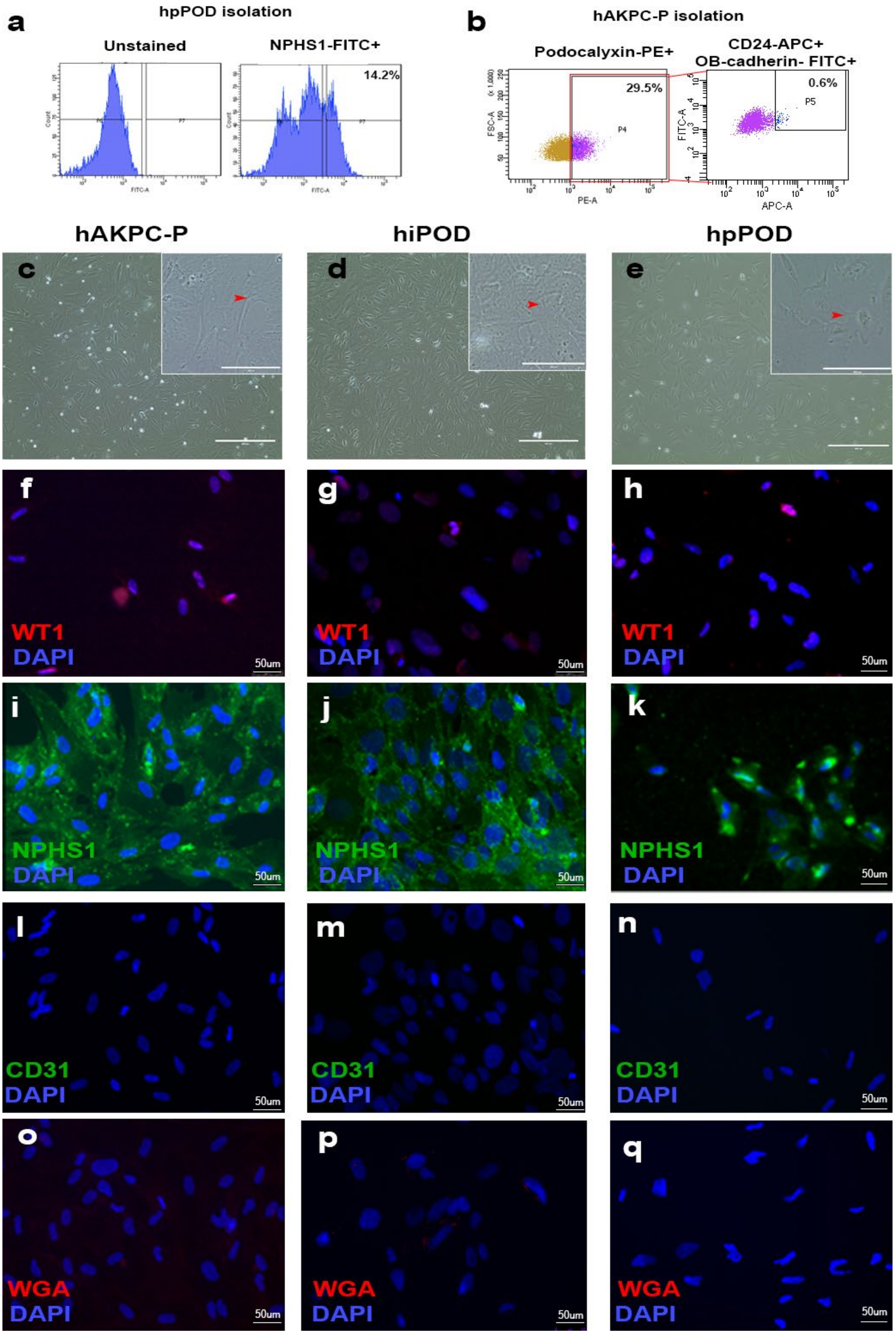
a
hpPOD isolation
Unstained
NPHS1-FITC+
14.2%
b
hAKPC-P isolation
Podocalyxin-PE+
29.5%
CD24-APC+
OB-cadherin- FITC+
0.6%
P4
P5
FSC-A
PE-A
FITC-A
APC-A
hAKPC-P
hiPOD
hpPOD
c
d
e
f
g
h
WT1
DAPI
i
j
k
NPHS1
DAPI
l
m
n
CD31
DAPI
o
p
q
WGA
DAPI
50um

Supplementary Figure 2. Characterization of podocyte lines.

a-b. Representative graph of FACS for hpPOD (**a**) and hAKPC-P (**b**). hpPOD were freshly isolated from a single cell suspension obtained after human glomeruli digestion based on the expression of nephrin (NPHS1-FITC, green). About 14.2% of the cells were positive for nephrin (**a**). hAKPC-P cells were isolated from human amniotic fluid total cell population based on co-expression of CD24 (APC, far red), OB-cadherin (FITC, green) and podocalyxin (PE, red) [25]. About 0.6-0.8% of the cells co-expressed the 3 markers (**b**).

c-e. Representative bright field imaging of hAKPC-P (**c**), hiPOD (**d**) and hpPOD (**e**) after 2 days of culture showing morphology of podocyte cells with presence of primary processes like structures (red arrow, scale bar= 50 μm, magnification scale bar= 400 μm). **f-q.** Representative immunofluorescence images for WT1 (Alexa555-red, **f-h**), for nephrin (NPHS1-FITC, green, **i-k**), for CD31 (FITC, green; **l-n**), and for WGA (Rhodamine, red; **o-q**) in hAKPC-P (**f,i,l,o**), in hiPOD (**g,j,m,p**) and in hpPOD (**h,k,n,q**). It is noticeable that all three podocyte lines are positive for podocyte markers, WT1 and nephrin, and negative for endothelial markers CD31 and WGA. Nuclei are stained with DAPI (blue). Scale bar = 50μm.

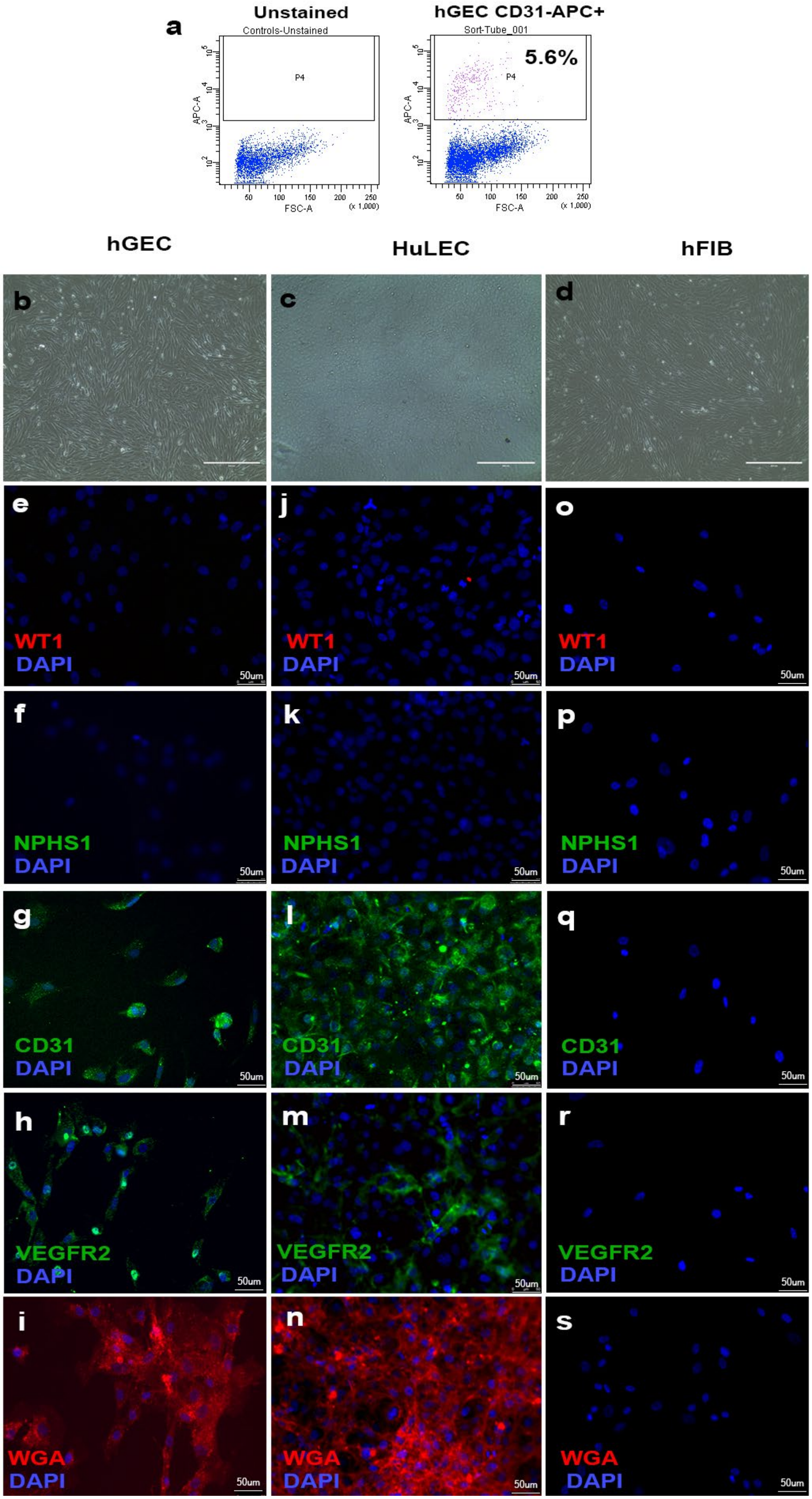
a
Unstained
Controls-Unstained
P4
APC-A
FSC-A
(x 1,000)
hGEC CD31-APC+
Sort-Tube_001
5.6%
hGEC
HuLEC
hFIB
b
c
d
e
WT1
DAPI
50um
j
o
f
NPHS1
k
p
g
CD31
l
q
h
VEGFR2
m
r
i
WGA
n
s

Supplementary Figure 3. Characterization of hGEC, HuLEC and hFIB.

a. Representative graph of FACS for hGEC showing isolation of CD31+ cells (Alexa-647, red) freshly isolated from single cell suspension obtained after human glomeruli digestion. About 5-6% of the cells were found positive for the endothelial marker. **b-d.** Representative bright field imaging of hGEC (**b**), HuLEC (**c**) and hFIB (**d**) after 2 days of culture (scale bar= 400 µm). Representative confocal images for WT1 (Alexa-555, red; **e**), for nephrin (NPHS1-FITC, green; **f**), for CD31 (FITC-green, **g**), for VEGFR2 (FITC-green, **h**), and for WGA (Rhodamine, red, **i**) in hGEC. As shown hGEC are negative for podocyte markers WT1 and nephrin, while they present positive expression of endothelial markers CD31, VEGFR2 and WGA. Nuclei are stained with DAPI (blue). Scale bar= 50µm. **j-n.** Representative confocal images for WT1 (Alexa-555, red; **j**), for nephrin (NPHS1-FITC, green; **k**); for CD31 (FITC, green; **l**), for VEGFR2 (FITC, green; **m**), and for WGA (Rhodamine, red; **n**) in HuLEC. As shown these cells represent a similar pattern of expression to hGEC; HuLEC are negative for podocyte markers WT1 and nephrin, while they present positive expression of endothelial markers like CD31, VEGFR2 and WGA. Nuclei are stained with DAPI (blue). Scale bar= 50µm. **o-s**. Representative confocal images for WT1 (Alexa-555, red; **o**), for nephrin (NPHS1-FITC, green; **p**); for CD31 (FITC, green; **q**), for VEGFR2 (FITC, green; **r**), and for WGA (Rhodamine, red; **s**) in HuLEC. As expected, hFIB are negative for both podocyte and endothelial markers. Nuclei are stained with DAPI (blue). Scale bar= 50µm.

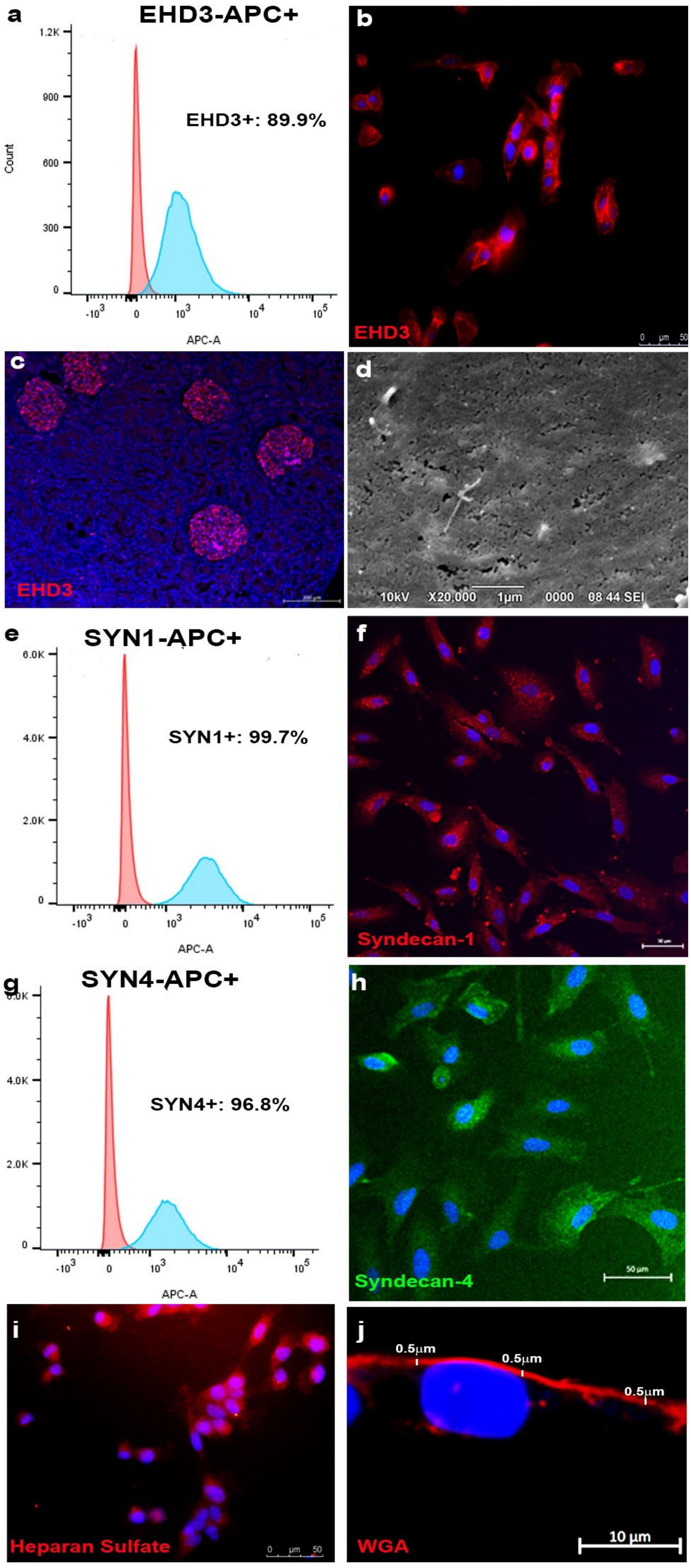

a
EHD3-APC+
EHD3+: 89.9%
Count
APC-A
b
EHD3
c
EHD3
d
10kV X20,000 1μm 0000 08 44 SEI
e
SYN1-APC+
SYN1+: 99.7%
APC-A
f
Syndecan-1
g
SYN4-APC+
SYN4+: 96.8%
APC-A
h
Syndecan-4
50 μm
i
Heparan Sulfate
j
0.5μm
0.5μm
0.5μm
WGA
10 μm

Supplementary Figure 4. In depth characterization of hGEC: glomerular origin, fenestrations, glycocalyx

a-c. Expression of EHD3 in hGEC. **a.** Representative graph of flow cytometry showing expression of EHD3 (Alexa-647, red) in freshly isolated hGEC. About 89.9% of the cells were found positive for the glomerular endothelial marker. **b.** Representative confocal image for EHD3 (Alexa-555, red) in freshly isolated hGEC. Nuclei are stained with DAPI (blue). Scale bar= 50µm. **c.** Representative confocal images for EHD3 (Alexa-555, red) in human adult kidney tissue. Expression of EHD3 is limited to the glomeruli, confirming the specificity of the marker to identify glomerular endothelial cells. Nuclei are stained with DAPI (blue). Scale bar= 300µm. **d.** Scanning electron micrograph of hGEC, showing the presence of fenestrations averaging 61 nm in diameter. Scale bar=1 µm. **e-j.** Expression of glycocalyx components in hGEC. **e,g** Representative graph of flow cytometry showing expression of syndecan-1 (Alexa-647, red, **e,** 99.7%), syndecan-4 (Alexa-647, red, **g,** 96.8%) in freshly isolated hGEC. **f,h,i** Representative confocal images for Syndecan-1 (Alexa-555, red; **f**), Syndecan-4 (Alexa-488, green, **h**) and Heparan Sulfate (Alexa-555, red, **i**) in hGEC. Nuclei are stained with DAPI (blue). Scale bar= 50µm. **j.** Representative confocal images for WGA on hGEC seeded on GOAC, confirming the thickness of the glycocalyx averaging about 0.5µm. Nuclei are stained with DAPI (blue). Scale bar= 10µm.

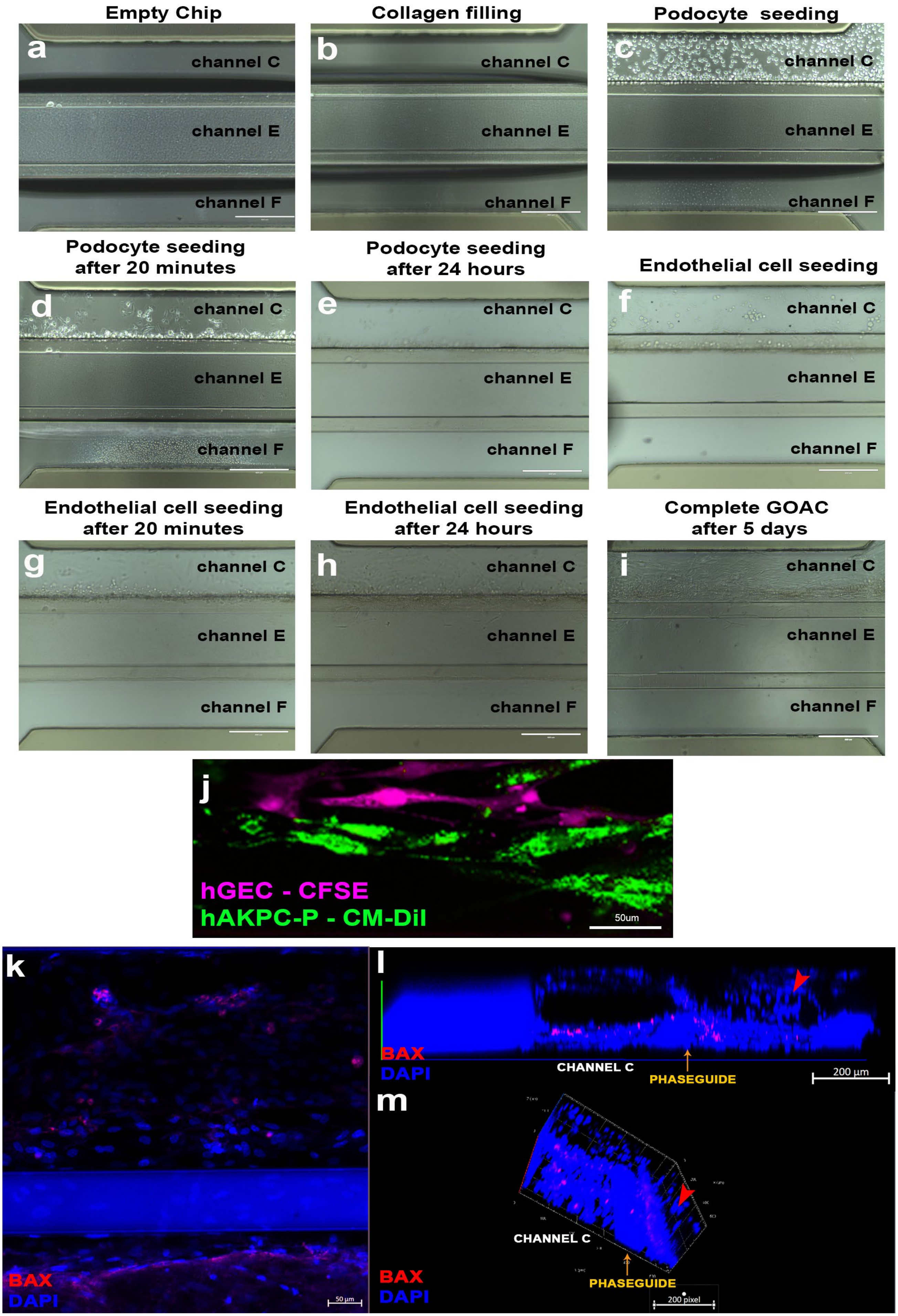
Empty Chip
Collagen filling
Podocyte seeding
a
b
c
channel C
channel E
channel F
Podocyte seeding
after 20 minutes
Podocyte seeding
after 24 hours
Endothelial cell seeding
d
e
f
Endothelial cell seeding
after 20 minutes
Endothelial cell seeding
after 24 hours
Complete GOAC
after 5 days
g
h
i
j
hGEC - CFSE
hAKPC-P - CM-Dil
50um
k
BAX
DAPI
50 µm
l
CHANNEL C
PHASEGUIDE
200 µm
m
200 pixel

Supplementary Figure 5. Description of the seeding process in Organoplates™

The barrier-free GOAC is created by progressive stages: first a layer of type I collagen enables podocytes (as example here hAKPC-P) to engraft; then hGEC are seeded in the same channel of podocytes and form capillary-like architecture; this interaction will initiate de novo generation of GBM. (**a**) Representative picture of an empty chip, prior to collagen application; (**b**) following collagen application and gelation (20 min at 37°C); (**c**) Immediately following seeding of hAKPC-P cells can be seen filling the top channel C; (**d**) 20 min following seeding of hAKPC-P, cells start layering onto the collagen layer; (**e**) 24hrs following hAKPC-P seeding, cells have fully engrafted on top of the collagen layer; (**f**) immediately following seeding of hGEC (48hrs after the initial seeding of hAKPC-P), cells start filling top channel C; (**g**) 20 min after the seeding of hGEC, cells start layering on top of the hAKPC-P; (**h**) 24hrs following hGEC seeding, cells have formed a layer; (**i**) 5 days following hGEC seeding the complete barrier is established. hGEC form a capillary-like structure in channel C. Experimental procedure described above for hAKPC-P and hGEC has been applied to every experiment described in this Manuscript, regardless of cell line. **j**. Representative confocal image of hAKPC-P labeled with CM-DiI (green, surface labeling) and hGEC-labeled with CFSE (magenta, surface labeling) after 7 days in culture on the chip. The formation of two layers and hAKPC-P and hGEC is clearly distinguishable. Scale bar: 50µm. **k-m** Representative confocal images of GOAC chip after 28 days in culture for apoptotic marker BAX (Alexa-555 red). Sporadic apoptotic cells can be seen in channel C as well as along the barrier. Passage in channel E by cells (red arrow) is evident by 3D reconstruction of Z-stack confocal images (**l,m**). Over-growth in channel E occurred only at 28 days and was not observed at prior time-points. Nuclei are stained with DAPI (blue). Scale bar= 50µm (**k**), 200 µm (**l, m**).

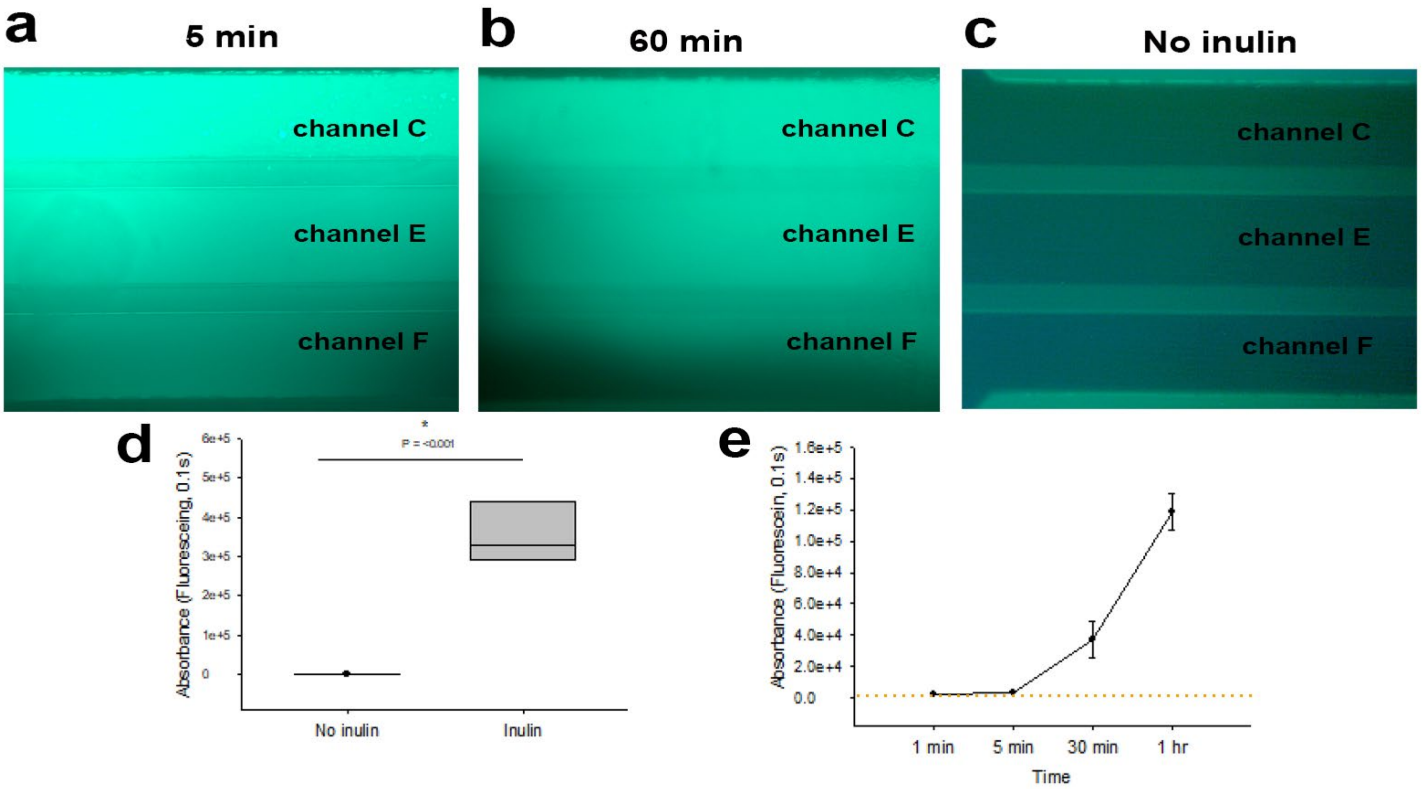

Supplementary Figure 6: Evaluation of permselectivity: inulin diffusion and albumin transwell comparison. **a-d.** Representative bright field showing inulin-FITC diffusion (cyan) after 5min (left column) and 60 min (right column) in hAKPC-P+hGEC chip, confirming free diffusion of inulin across the hAKPC-P+hGEC barrier. Birght field image of hAKPC-P+hGEC chip without inulin shown for comparison (**c**). **d.** Box plot graph of fluorescein absorbance in filtrate collected from channel F after 60 min following incubation with inulin. P<0.001. Significant differences were determined by a one-way ANOVA and Holm-Sidak post hoc test. Significant differences were determined by a one-way ANOVA and Holm-Sidak post hoc test Box plots show the median, the 25th and 75th percentiles, whiskers (median ± 1.5 times interquartile range), and outliers (•) **e.** Graph of fluorescein absorbance in filtrate collected after 1, 5, 30 and 60 min following incubation with albumin-FITC (40mg/ml) from the lower chamber of hpPOD+hGEC barriers generated on transwells (see Methods for more details). Orange dotted line represents the average leakage on GOAC after 60 minutes. Variation expressed as standard error.

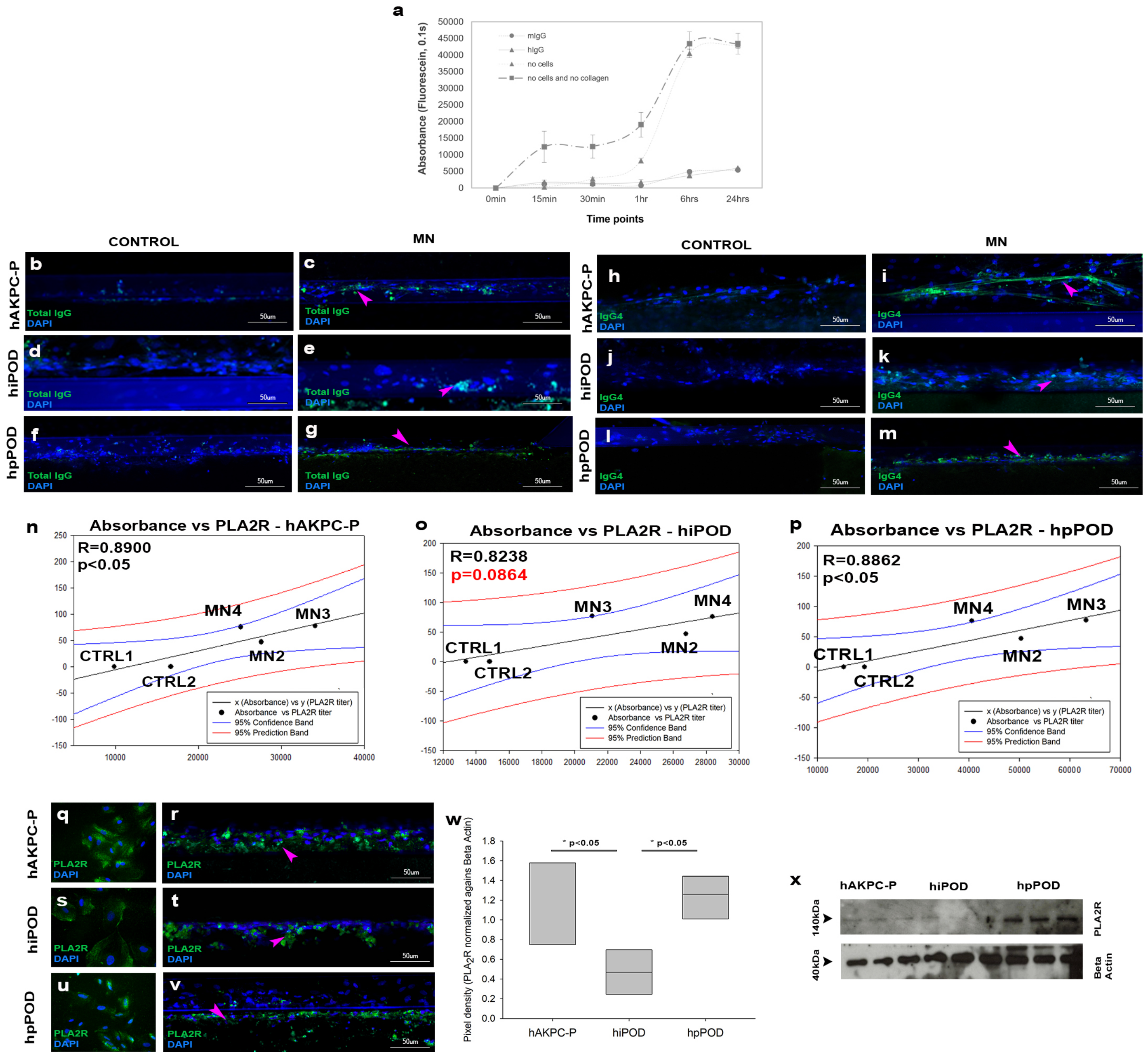

Supplementary Figure 7: Evaluation of IgG ability to cross the hGEC monolayer, expression of total IgG, IgG4, and PRLA$_2$R in podocyte lines and correlation of GOAC to anti-PLA2R autoantibody concentration.

a. Evaluation of IgG ability to cross the glomerular endothelial barrier formed on a transwell. Following formation of a continuous monolayer, 1mg/ml FITC-labeled human and mouse IgG were added to the top chamber. Measure of fluorescein absorbance was performed on 50μl media collected on the bottom chamber at different time points (0m, 5m, 15m, 1hr, 6hrs, 24hrs). Negative controls were experiments performed 1) devoid of cells or 2) devoid of cells and without collagen I coating. **b-g.** Representative confocal image for expression of total IgG (FITC-green) in hAKPC-P+GEC chip (**b-c**), in hiPOD+hGEC chip (**d-e**), and

hpPOD+hGEC chip (**f-g**) after exposure to 0.5% of serum from healthy controls for 24hrs (**b,d,f**) and after exposure of 0.5% serum from MN patients for 24hrs (**c,e,g**). All MN chips show expression of total IgG, see magenta arrow. **h-m**. Representative confocal image for expression of IgG4 (FITC-green) in hAKPC-P+GEC chip (**h,i**), in hiPOD+hGEC chip (**j,k**), and hpPOD+hGEC chip (**l,m**) after exposure to 0.5% of serum from healthy controls for 24hrs (**h,j,l**) and after exposure of 0.5% serum from MN patients for 24hrs (**i,k,m**). All MN chips show expression of IgG4, see magenta arrow. **n-p.** Correlation between proteinuria on GOAC and patient's PLA_2R titer. Relationship between hAKPC-P+hGEC chip proteinuria for CTRL1-2, MN2-4 and corresponding PLA_2R titer suggesting a very strong correlation between serum concentration of anti-PLA_2R antibody and response in the chip (measured as albumin leakage). R: 0.8900, $P<0.05$. Regression analysis. Equation: Polynomial, linear. Blue lines = 95% Confidence Band; Red lines = 95% Prediction Band. PLA_2R Titer: MN2: 47; MN3:77.2; MN4: 75.6. (**n**). Relationship between hiPOD+hGEC chip proteinuria for CTRL1-2, MN2-4 and corresponding PLA_2R titer suggesting a weak correlation between serum concentration of anti-PLA_2R antibody and response in the chip (measured as albumin leakage). R: 0.8238, not significant. Regression analysis. Equation: Polynomial, linear. Blue lines = 95% Confidence Band; Red lines = 95% Prediction Band PLA_2R Titer: MN2: 47; MN3:77.2; MN4: 75.6. (**o**). Relationship between hpPOD+hGEC chip proteinuria for CTRL1-2, MN2-4 and corresponding PLA_2R titer suggesting a very strong correlation between serum concentration of anti-PLA_2R antibody and response in the chip (measured as albumin leakage). R:0.8862, $P<0.05$. Regression analysis. Equation: Polynomial, linear. Blue lines = 95% Confidence Band; Red lines = 95% Prediction Band PLA_2R Titer: MN2: 47; MN3:77.2; MN4: 75.6. (**p**)**. q-v**. Representative confocal image for expression of PLA_2R (FITC-green) in hAKPC-P (**q**), in hiPOD (**s**), and hpPOD (**u**) in culture after exposure to 0.5% of serum from healthy controls for 24hrs and after seeding on the chip together with hGEC (**r,t,v**). All three podocyte lines show expression of PLA_2R on their surface, arrow. Nuclei are stained with DAPI (blue). All pictures: scale bar = 50 µm, except **r,t,v**: scale bar = 50 µm. **w-x.** Western Blot analysis for PLA_2R (140kDA) and beta actin (40kDa) in hAKPC-P+hGEC, hiPOD+hGEC and hpPOD+hGEC GOACs confirmed expression for PLA_2R by all three chips (**x**). Quantification of PLA_2R expression was performed by measuring pixel density and followed by normalization against beta actin (**w**). Significant differences were determined by a one-way ANOVA and Student-Newman-Keuls post hoc test $^*P<0.05$. Number of replicates per experimental group: 3. Box plots show the median, the 25th and 75th percentiles, whiskers (median ± 1.5 times interquartile

range), and outliers (•) Western blot images were cropped to show the relevant bands and improve clarity. Full gel images available in the supplementary material.

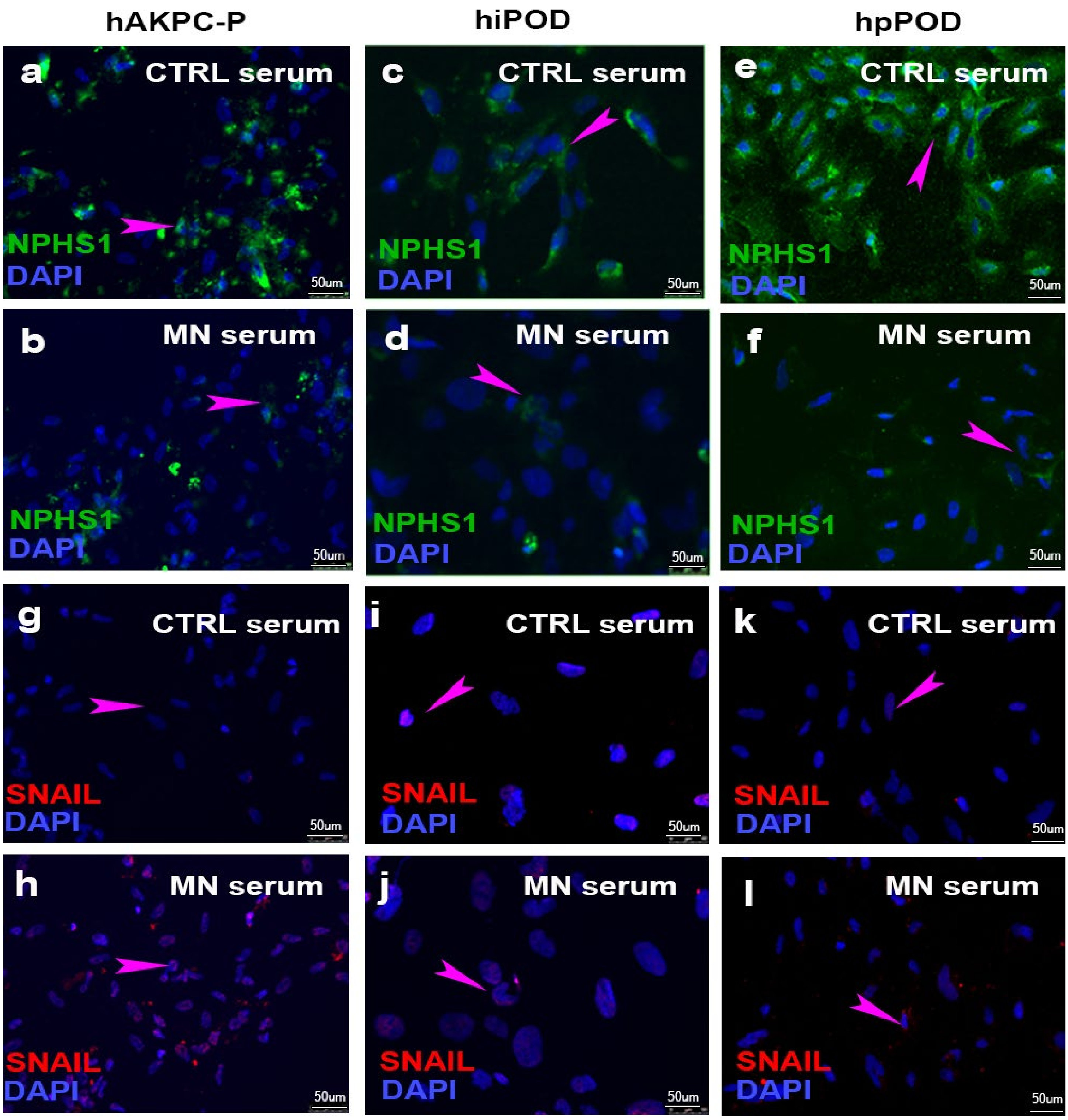

hAKPC-P
hiPOD
hpPOD
a
CTRL serum
NPHS1
DAPI
50um
b
MN serum
NPHS1
DAPI
50um
c
CTRL serum
NPHS1
DAPI
50um
d
MN serum
NPHS1
DAPI
50um
e
CTRL serum
NPHS1
DAPI
50um
f
MN serum
NPHS1
DAPI
50um
g
CTRL serum
SNAIL
DAPI
50um
h
MN serum
SNAIL
DAPI
50um
i
CTRL serum
SNAIL
DAPI
50um
j
MN serum
SNAIL
DAPI
50um
k
CTRL serum
SNAIL
DAPI
50um
l
MN serum
SNAIL
DAPI
50um

Supplementary Figure 8: Mechanism of action in membranous nephropathy

a-f. Representative immunofluorescence images for expression of nephrin (NPHS1-FITC, green) in hAKPC-P (**a-b**), in hiPOD (**c-d**), and hpPOD (**e-f**) after exposure to 0.5% of serum from healthy individuals for 24hrs (**a,c,e**) and after exposure of 0.5% serum from MN patients for 24hrs (**b,d,f**). Expression of nephrin (magenta arrow) is decreased in all three lines, thus confirming that exposure of serum from NM causes damage to podocytes and loss of slit diaphragm protein. Nuclei are stained with DAPI (blue). All pictures: scale bar = 50 μm. **g-l.** Representative immunofluorescence images for expression of snail (FITC, green) in hAKPC-P (**g-h**), in hiPOD (**i-j**), and hpPOD (**k-l**) after exposure to 0.5% of serum from healthy individuals for 24hrs (**g,i,k**) and after exposure of 0.5% serum from MN patients for 24hrs (**h,j,l**). Expression of nuclear snail (magenta arrow) is evident in all three lines, thus suggesting that exposure of serum from NM increases activation of this signaling. All pictures: scale bar = 50 μm.

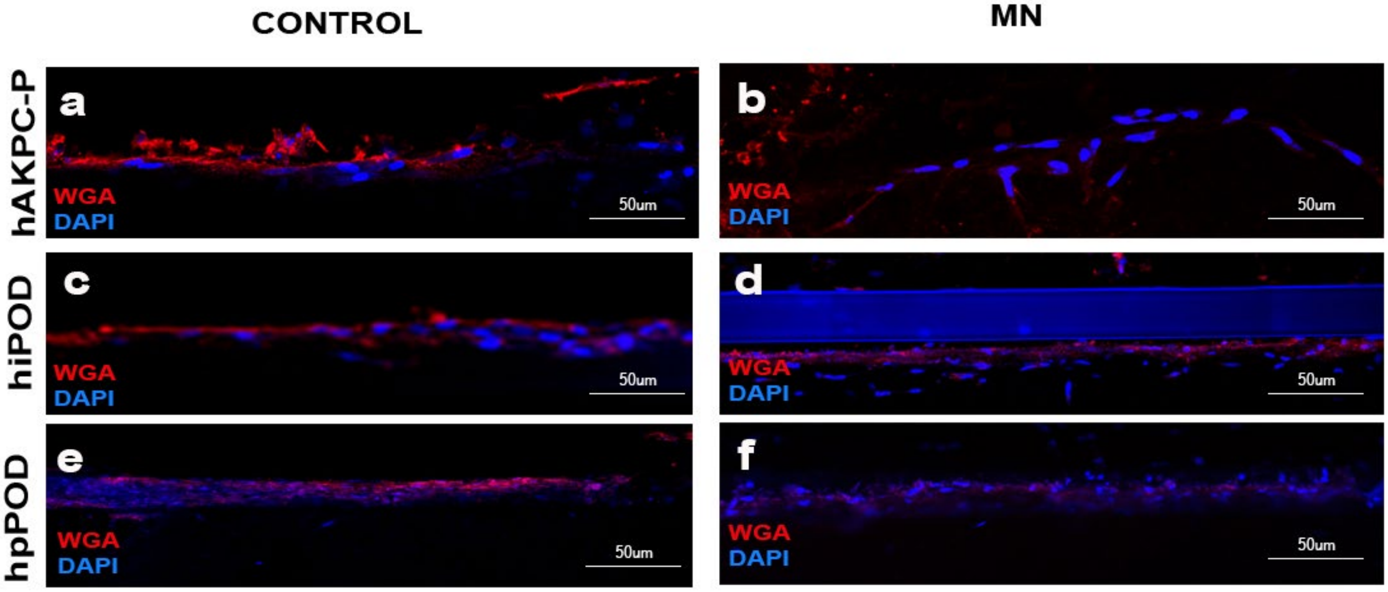

Supplementary Figure 9: Membranous nephropathy induced damage in endothelial cells

a-f. Representative confocal image for expression of WGA (Rhodamine, red) in hAKPC-P+GEC chip (**a-b**), in hiPOD+hGEC chip (**c-d**), and hpPOD+hGEC chip (**e-f**) after exposure to 0.5% of serum from healthy controls for 24hrs (**a,c,e**) and after exposure of 0.5% serum from MN patients for 24hrs (**b,d,f**). All chips show decrease of WGA expression (damage to the glycocalyx) after exposure to MN serum, thus confirming that also hGEC present MN-induced damage. Nuclei are stained with DAPI (blue). All pictures: scale bar = 50 μm.

Gating Strategy

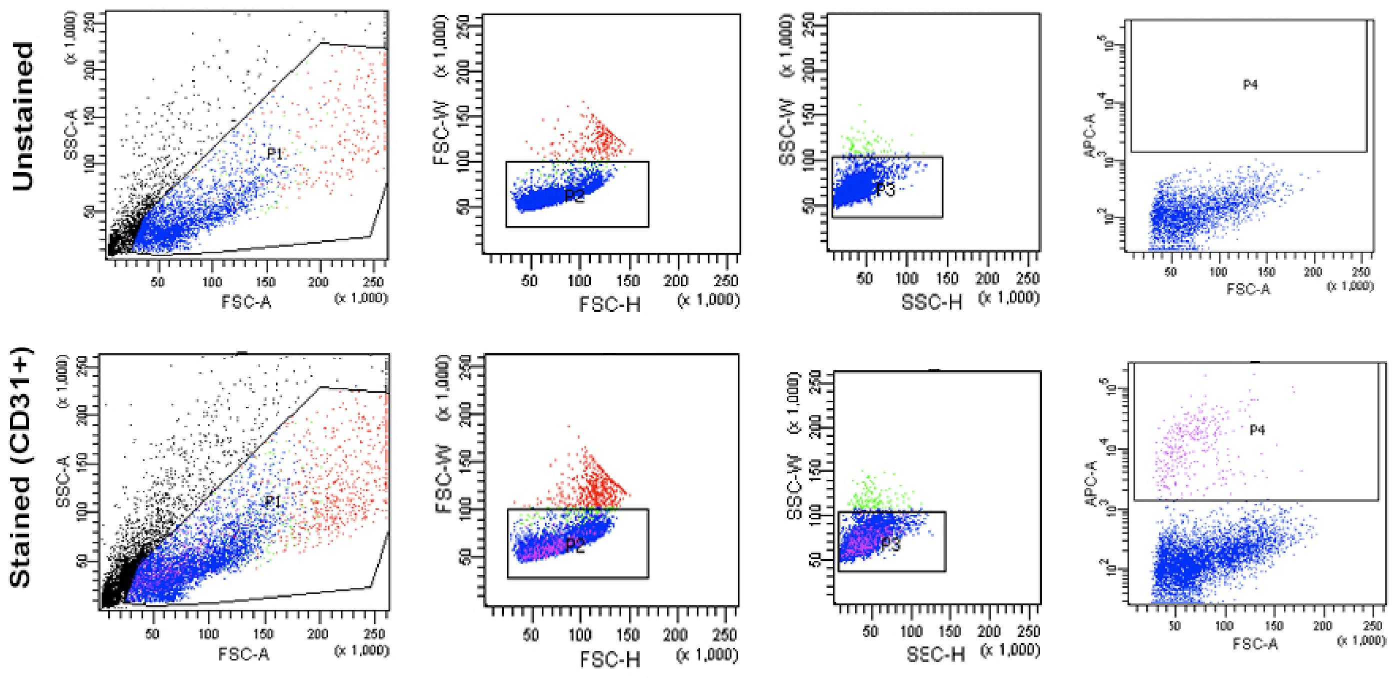

Supplementary Figure 10: Gating strategy

Representative plots showing gating strategy as performed for FACS isolation and flow cytometry analysis for the isolation of CD31+ hGEC (Supplementary Figure 3a). Live cells were first gated based on forward (FSC) and side scatter (SSC) and dead cells were excluded from the analysis. Further gating was performed to remove duplets based on FSC-W/FSC-H and SSC-W/SSC-H. Gating for positive cells (in this case CD31) was performed to exclude all events occurring in unstained cells for each channel (Alexa-488/FITC, APC, PE – fluorochrome dependent on the experiment). Gating was performed following the same criteria but independently for each sample to reflect differences between the analyzed populations. Same gating strategy was used for all flow cytometry and/or FACS presented in the current Manuscript.

Supplementary Figure 11: Original blot images for COL4A3 and LAMA5 on hAKPC+hGEC, hiPOD +hGEC, hpPOD+hGEC GOAC - Corresponding to blots in Figure 2w-y

In order: LAMA5, COL4A3 and BETA ACTIN for hAKPC GOAC; LAMA5, COL4A3 and BETA ACTIN for iPOD and pPOD GOAC. **COL4A3 was measured around 50 kDa, Laminin a5 was measured below 70 kDA, B-actin around 40kDa**

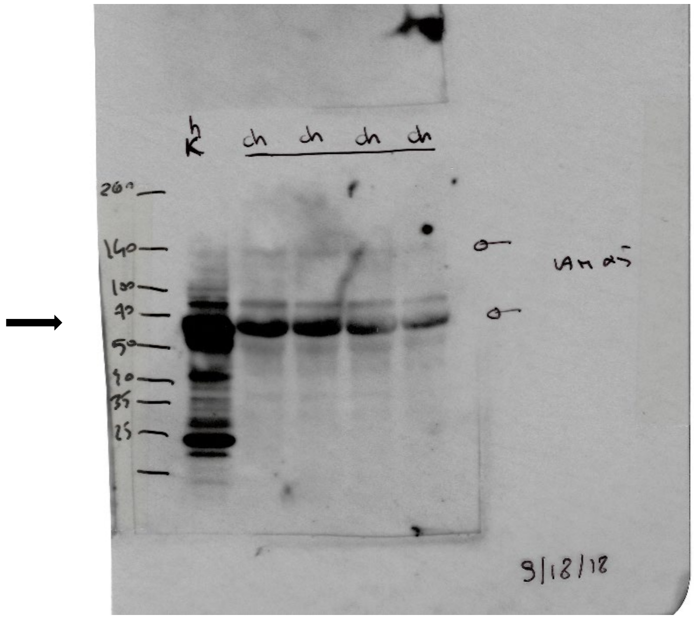

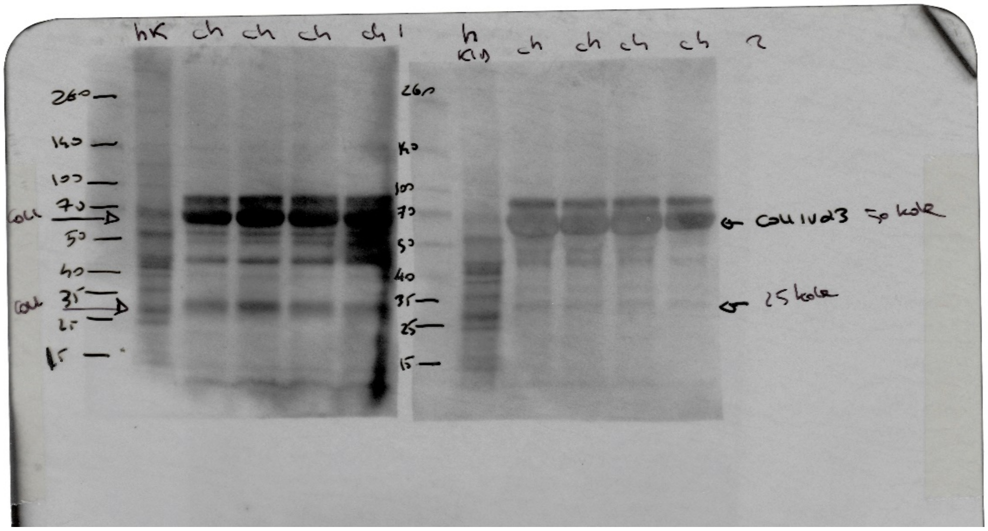

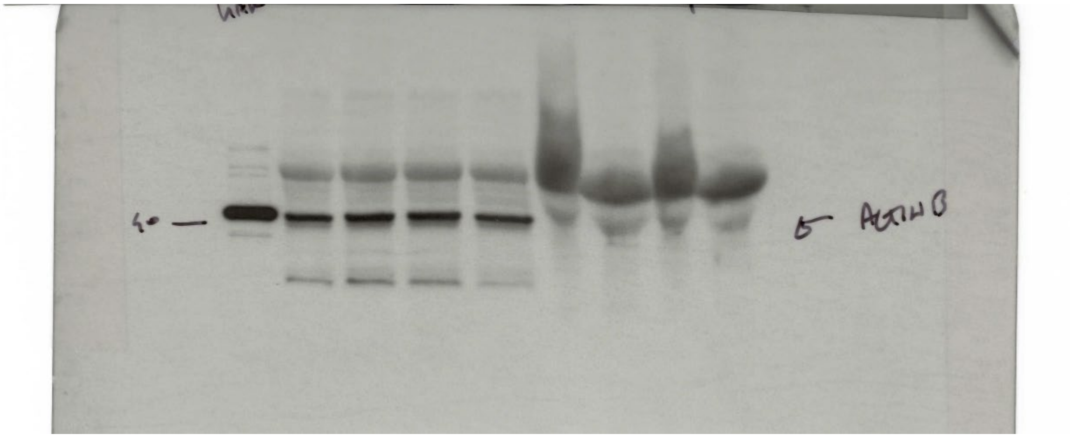
Actin B

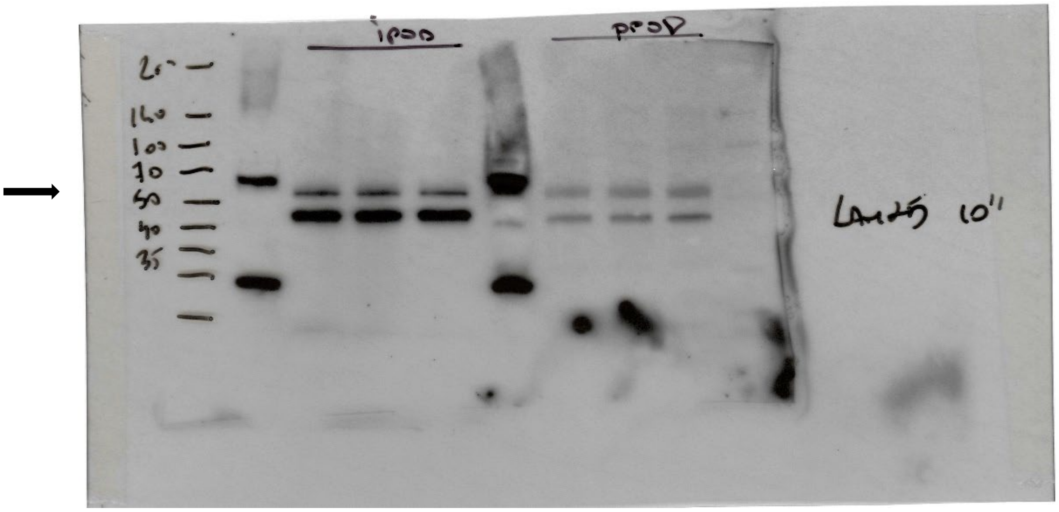
ipod
140
100
70
50
40
35

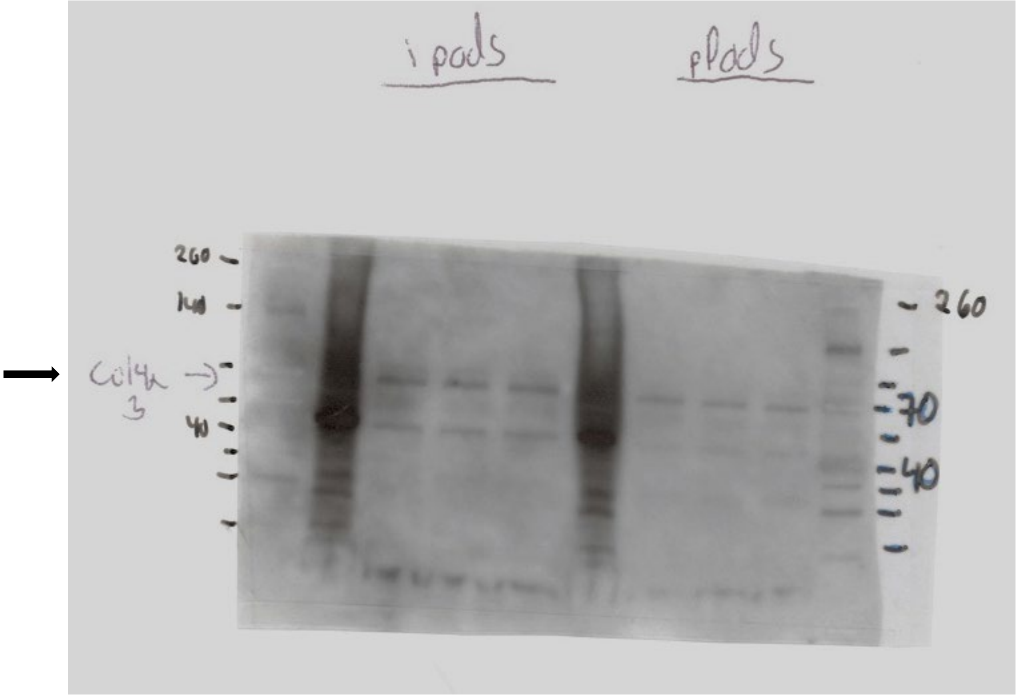
ipods
260
140
40
260
70
40

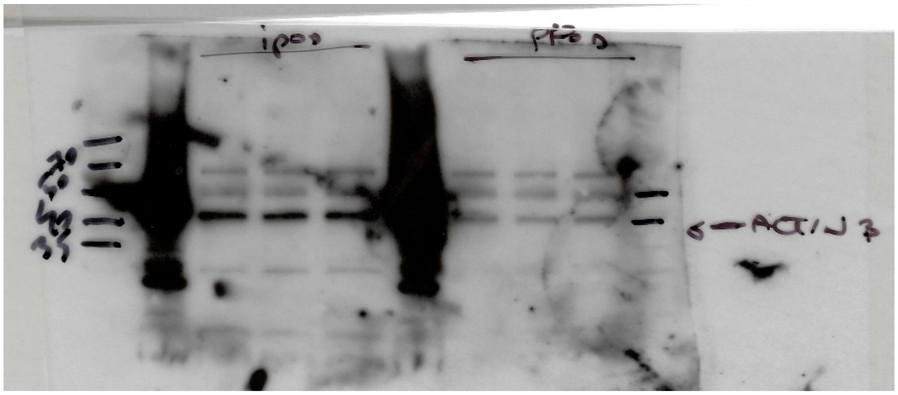

Supplementary Figure 12: Original blot images for PLA2R on hAKPC, hiPOD, hpPOD corresponding to blots in Figure S7. PLA2R was measured around 140 kDa

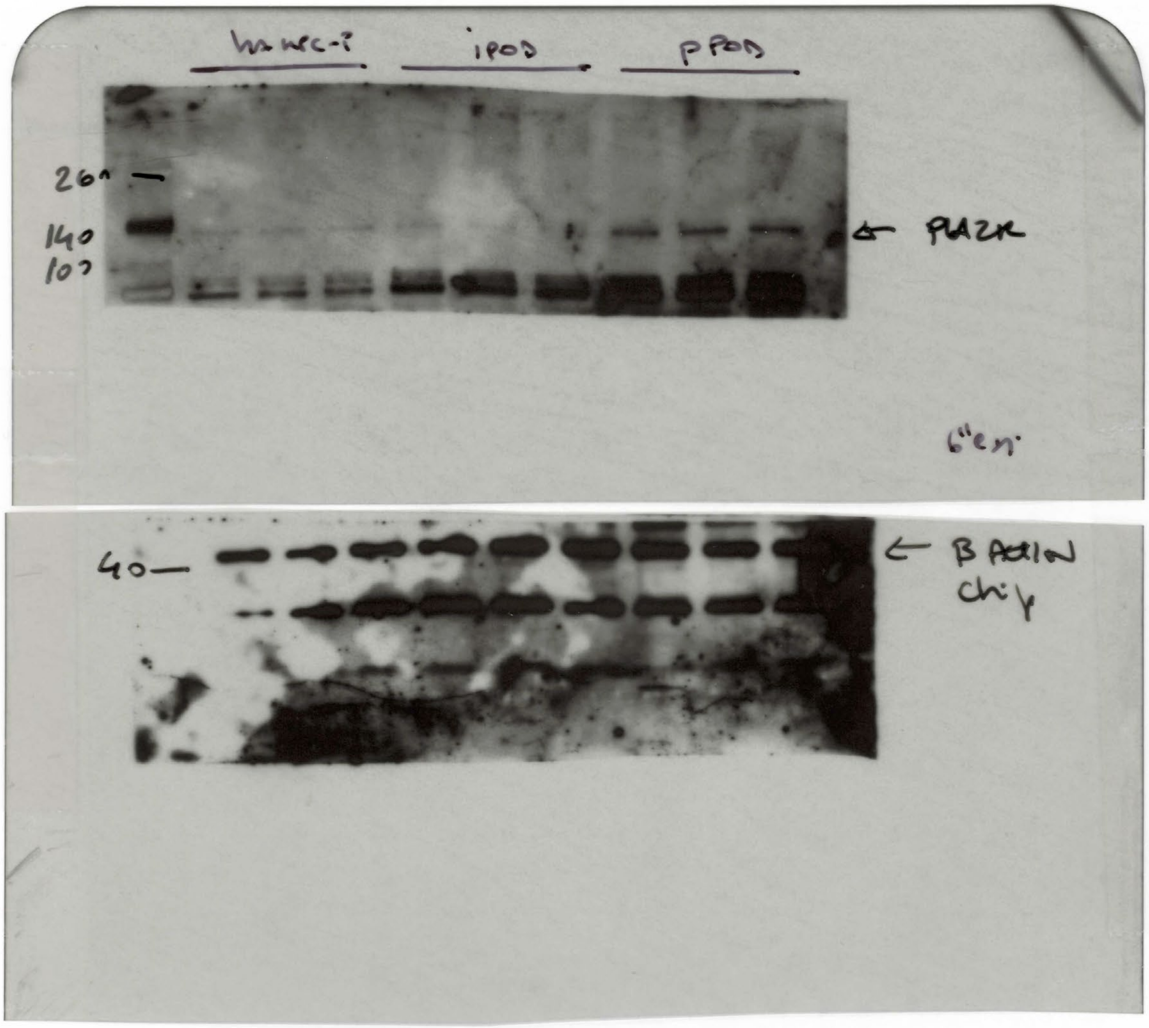

Supplementary Figure 13 : Original blot images for C3d on hpPOD, hiPOD, and hAKPC, cells culture with either control or MN patient serum. Measured below 140 kDa. Corresponding to blots in Figure 6f

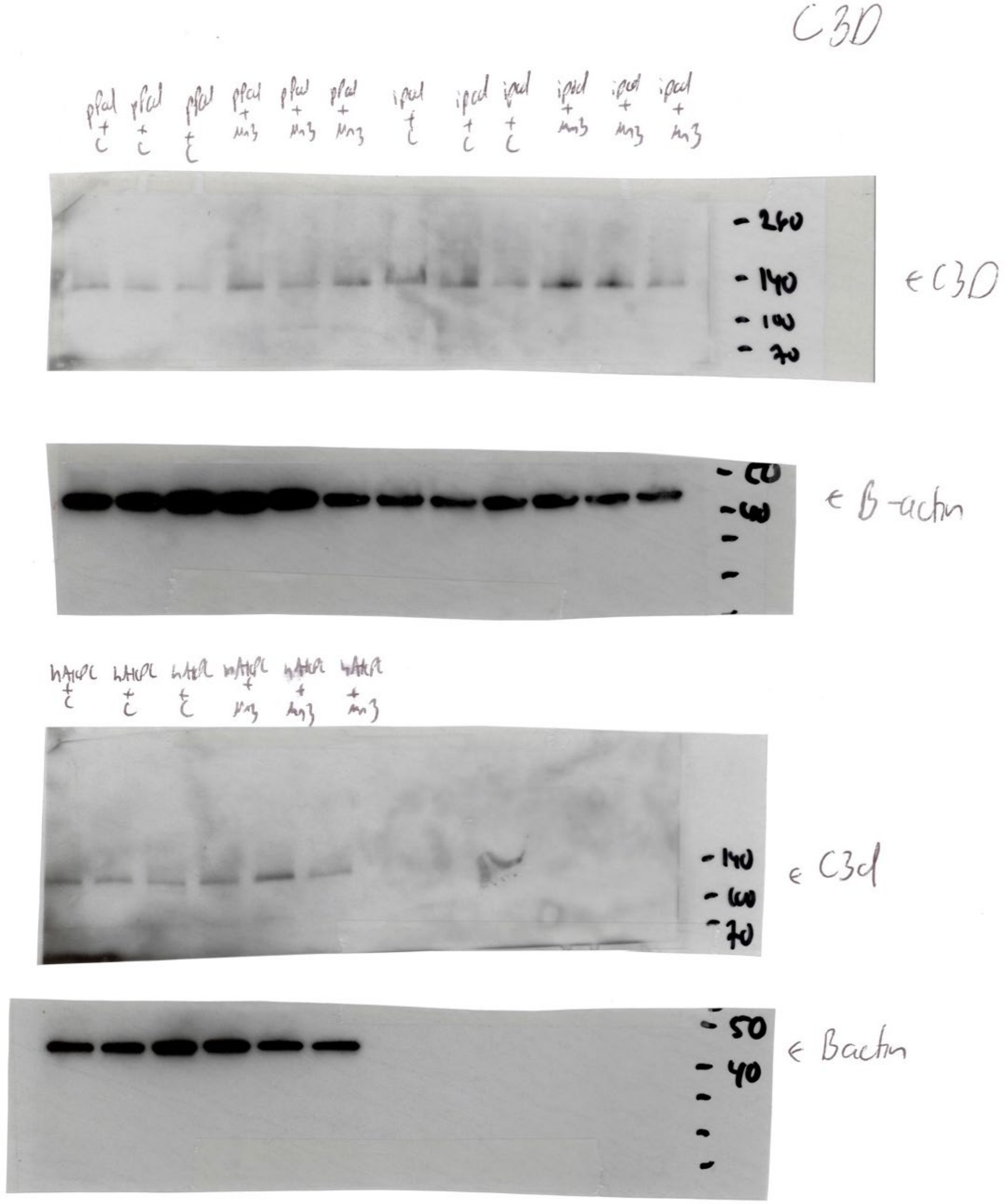

Supplementary Figure 14: Original blot images for NPHS1 on hAKPC+hGEC, hiPOD+hGEC, hpPOD+hGEC GOAC Corresponding to blots in Figure 6h Measured around 138 kDa.

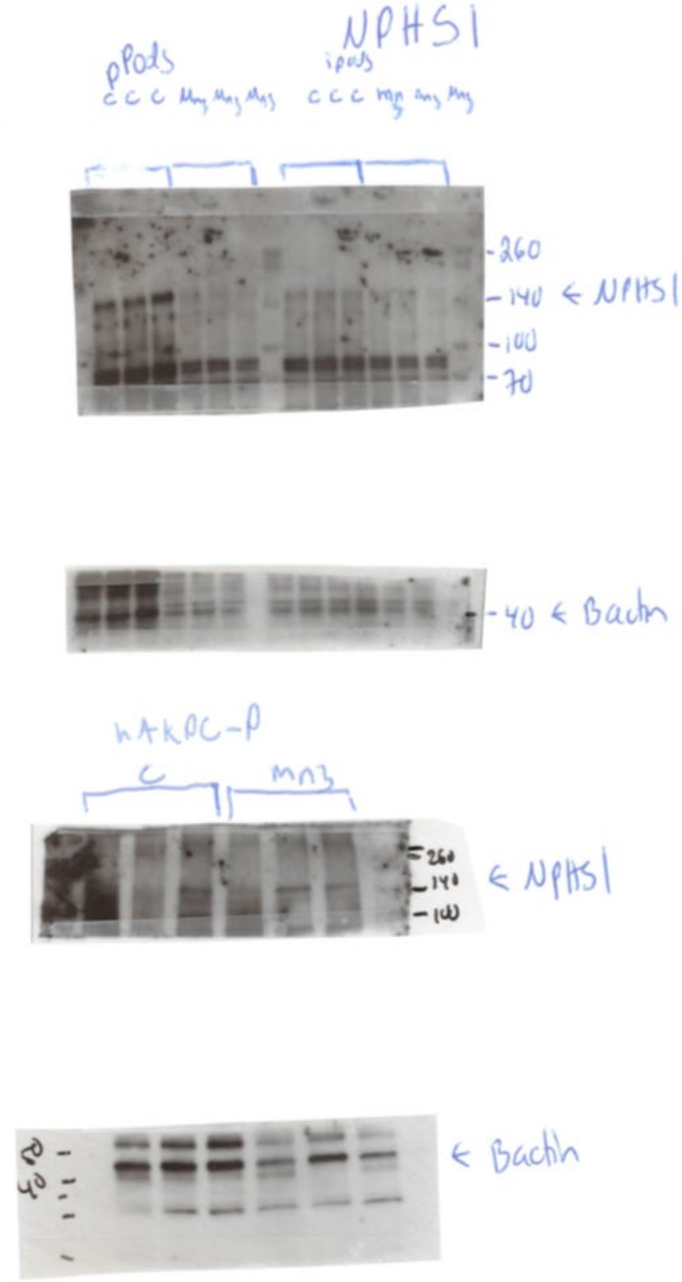

Supplementary Tables

SUPPLEMENTARY TABLE 1 - SUMMARY OF NUMBER OF CHIPS USED FOR PERMSELECTIVITY								
GOAC Basal Permselectivity								
hAKPC-P +hGEC	hiPOD +hGEC	hpPOD +hGEC	hAKPC-P +HuLEC	hFIB +hGEC	no cell			
12	6	7	13	19	3			
Long term experiment								
hAKPC-P+hGEC					hpPOD+hGEC			
7d	14d	21d	28d		7d	14d	21d	28d
13	10	9	4		7	22	15	15
hiPOD+hGEC								
7d	14d							
10	11							
Puromycin Aminonucleoside damage								
hAKPC-P +hGEC	hAKPC-P +hGEC + PAN	hiPOD +hGEC	hiPOD +hGEC + PAN	hpPOD +hGEC	hpPOD +hGEC + PAN			
12	4	6	4	7	3			
Membranous Nephropathy (MN) model								
hAKPC-P+hGEC GOAC								
CTRL1	CTRL2	MN1	MN2	MN3	MN4	MN5	MN6	
7	8	4	7	3	4	7	3	
hiPOD+hGEC GOAC								
CTRL1	CTRL2	MN1	MN2	MN3	MN4	MN5	MN6	
6	4	11	5	4	11	5	4	
hpPOD+hGEC GOAC								
CTRL1	CTRL2	MN1	MN2	MN3	MN4	MN5	MN6	
8	9	6	7	7	6	7	7	
Diabetic Nephropathy model								
10mM	15mM	20mM						
16	4	7						
CKD models								
CTRL1	CTRL2	FSGS1	FSGS2	FSGS3	AS	PKD1	PKD2	PKD3
7	8	9	3	4	4	5	5	3
α-MSH effect on MN					Alport Syndrome – Disease modeling			
CTRL2	MN3	MN3 + a-MSH			hAKPC-P +hGEC		AS-hAKPC-P +hGEC	
6	9	11			12		17	
