# Supplementary material for: A glomerulus-on-a-chip to recapitulate the human glomerular filtration barrier
Source: Nat Commun. 2019 Aug 13;10:3656. doi: 10.1038/s41467-019-11577-z (PMC6692336; doi:10.1038/s41467-019-11577-z)
Supplement: Supplementary file 3 — Reporting Summary [file 41467_2019_11577_MOESM3_ESM.pdf]

nature research

Corresponding author(s): Stefano Da Sacco, Laura Perin

Reporting Summary

Nature Research wishes to improve the reproducibility of the work that we publish. This form provides structure for consistency and transparency in reporting. For further information on Nature Research policies, see Authors & Referees and the Editorial Policy Checklist.

Statistical parameters

When statistical analyses are reported, confirm that the following items are present in the relevant location (e.g. figure legend, table legend, main text, or Methods section).

n/a	Confirmed	
☐	☒	The exact sample size (n) for each experimental group/condition, given as a discrete number and unit of measurement
☐	☒	An indication of whether measurements were taken from distinct samples or whether the same sample was measured repeatedly
☐	☒	The statistical test(s) used AND whether they are one- or two-sided<br>*Only common tests should be described solely by name; describe more complex techniques in the Methods section.*
☐	☒	A description of all covariates tested
☐	☒	A description of any assumptions or corrections, such as tests of normality and adjustment for multiple comparisons
☐	☒	A full description of the statistics including central tendency (e.g. means) or other basic estimates (e.g. regression coefficient) AND variation (e.g. standard deviation) or associated estimates of uncertainty (e.g. confidence intervals)
☒	☐	For null hypothesis testing, the test statistic (e.g. F, t, r) with confidence intervals, effect sizes, degrees of freedom and P value noted<br>*Give P values as exact values whenever suitable.*
☒	☐	For Bayesian analysis, information on the choice of priors and Markov chain Monte Carlo settings
☒	☐	For hierarchical and complex designs, identification of the appropriate level for tests and full reporting of outcomes
☐	☒	Estimates of effect sizes (e.g. Cohen's d, Pearson's r), indicating how they were calculated
☐	☒	Clearly defined error bars<br>*State explicitly what error bars represent (e.g. SD, SE, CI)*

Our web collection on statistics for biologists may be useful.

Software and code

Policy information about availability of computer code

Data collection	NA
Data analysis	NA

For manuscripts utilizing custom algorithms or software that are central to the research but not yet described in published literature, software must be made available to editors/reviewers upon request. We strongly encourage code deposition in a community repository (e.g. GitHub). See the Nature Research guidelines for submitting code & software for further information.

Data

Policy information about availability of data

All manuscripts must include a data availability statement. This statement should provide the following information, where applicable:

- Accession codes, unique identifiers, or web links for publicly available datasets
- A list of figures that have associated raw data
- A description of any restrictions on data availability

The authors declare that [the/all other] data supporting the findings of this study are available within the paper [and its supplementary information files].

Field-specific reporting

Please select the best fit for your research. If you are not sure, read the appropriate sections before making your selection.

☒ Life sciences ☐ Behavioural & social sciences ☐ Ecological, evolutionary & environmental sciences

For a reference copy of the document with all sections, see nature.com/authors/policies/ReportingSummary-flat.pdf

Life sciences study design

All studies must disclose on these points even when the disclosure is negative.

Sample size	To reach statistical significance at least 3 replicates were used for each experiment.
Data exclusions	No endpoint data were excluded from the analysis.
Replication	To verify reproducibility of the current work, experiments were performed in replicates. Same cohort of experiment was performed at different times and by different lab members to ensure unbiased results.
Randomization	No randomization was performed.
Blinding	Experiments were not subject to blinding

Reporting for specific materials, systems and methods

Materials & experimental systems

n/a	Involved in the study	
☐	☒	Unique biological materials
☐	☒	Antibodies
☐	☒	Eukaryotic cell lines
☒	☐	Palaeontology
☒	☐	Animals and other organisms
☐	☒	Human research participants

Methods

n/a	Involved in the study	
☒	☐	ChIP-seq
☐	☒	Flow cytometry
☒	☐	MRI-based neuroimaging

Unique biological materials

Policy information about availability of materials

Obtaining unique materials	No unique material was used for the current experiment. All cell lines are either widely available or their isolation method has been published previously or within this Manuscript.

Antibodies

Antibodies used	List of antibodies, including company, catalogue number, concentration for the different assays is reported in the Methods section
Validation	Antibodies were chosen for each assay based on the validation reported by each manufacturer on their publicly accessible website and corresponding data-sheets. Further validation of antibody specificity for immunofluorescence staining was performed by running parallel appropriate controls, including samples stained with secondary antibody in the absence of primary antibody. Validation of antibody specificity for FACS was confirmed by staining isolated cells for other cell specific markers (i.e. WT1, nephrin for podocytes; CD31, VEGFR2, WGA for endothelial cells). Validation of antibodies for WB was performed by running appropriate positive controls whenever appropriate (COL4A3) or indirectly validated by confirming immunofluorescence results on the same cell line.

Eukaryotic cell lines

Policy information about cell lines

Cell line source(s)	hAKPC-P were derived by discarded human amniotic fluid samples as described in Da Sacco et al., 2013.

Cell line source(s)	Human lung fibroblasts and HuLECs were obtained commerically as detailed in the Methods.<br>Human primary podocytes and human primary glomerular endothelial cells were freshly isolated from adult human kidneys as detailed in the Methods.<br>Human immortalized podocytes were kindly donated by Dr. Reiser as detailed in the Methods
Authentication	Authentication of each cell line was performed by immunofluorescence, flow cytometry and Western Blotting to confirm clel identity.
Mycoplasma contamination	Cells were not tested for Mycoplasma contamination.
Commonly misidentified lines (See ICLAC register)	NA

Human research participants

Policy information about studies involving human research participants

Population characteristics	Our study involved sera collected from healthy individuals and individuals with membranous nephropathy, focal segmantal glomerulosclerosis or polycystic kidney disease, or Alport syndrome. We had also access to unidentified amniotic fluid discarded tissues, kidney tissue.
Recruitment	Samples were obtained by Drs. Joaquin Manrique (Complejo Hospitalario de Navarra, Pamplona, Spain) and Andrea Angeletti (S. Orsola-Malpighi Hospital, University of Bologna, Bologna, Italy) from consenting subjects, according to IRB approved protocols at both centers. The samples of amniotic fluid were obtained from Genzyme. The sample of Alport Syndrome amniotic fluid was obtained from the Telethon Biobank (Italy). Kidney tissues were obtained from the transplant procurement system. All samples were de-identifed, obtained from consenting subjects and according to IRB approved protocols.

Flow Cytometry

Plots

Confirm that:

☒ The axis labels state the marker and fluorochrome used (e.g. CD4-FITC).

☒ The axis scales are clearly visible. Include numbers along axes only for bottom left plot of group (a 'group' is an analysis of identical markers).

☒ All plots are contour plots with outliers or pseudocolor plots.

☒ A numerical value for number of cells or percentage (with statistics) is provided.

Methodology

Sample preparation	FACS: cells were blocked using 1X human IgG (Sigma c# I2511) for 30 min then stained with the specified antibodies, 1 ug/1x106 cells/100 µl IgG solution unless otherwise specified on the datasheet, for 1 hour on ice. Cells were then washed twice in PBS and filtered immediately before sorting. Unstained and single positive controls were used to perform area scaling, exclude autofluorescence and perform fluorochrome compensation when needed.<br><br>For flow cytometry analysis, cells were fixed in 4% paraformaldehyde (Santa Cruz Biotechnology c# sc-281692) for 10 minutes and permeabilized with 0.05% saponin for nuclear proteins. Cells were then blocked in 1X human IgG solution for 10 minutes and incubated with the chosen antibody. A
Instrument	FACSAria
Software	Anysis was performed on a FACScanto machine using FACSDiva software. Gating strategy was performed as described above. Histogram plots were obtained using FlowJO software.
Cell population abundance	Analysis of post sort fraction was performed by fixing the cells in 4% PFA and performing flow cytometry analysis for relevant positive and negative markers.
Gating strategy	Gating strategy described in Figure S1. Live cells were first gated based on forward (FSC) and side scatter (SSC) and dead cells were excluded from the analysis. Further gating was performed to remove duplets based on FSC-W/FSC-H and SSC-W/SSC-H. Gating for positive cells (in this case CD31) was performed to exclude all events occurring in unstained cells for each channel (Alexa-488/FITC, APC, PE – fluorochrome dependent on the experiment). Gating was performed following the same criteria but independently for each sample to reflect differences between the analyzed populations.

☒ Tick this box to confirm that a figure exemplifying the gating strategy is provided in the Supplementary Information.
